# An unusual case report of mitral valve apparatus sparing left atrial appendage vegetation presenting as endogenous endophthalmitis

**DOI:** 10.1186/s43044-021-00176-3

**Published:** 2021-06-06

**Authors:** Rupesh Kumar, Vidur Bansal, Vikram Halder, Nirupan Sekhar Chakraborty, Krishna Prasad Gourav

**Affiliations:** 1 Department of Cardiothoracic and Vascular Surgery, Post Graduate Institute of Medical Education and Research, Chandigarh, 160012 India; 2 Department of Anesthesia, Post Graduate Institute of Medical Education and Research, Chandigarh, India

**Keywords:** Endophthalmitis, Vegetation, Infective endocarditis

## Abstract

**Background:**

Ocular manifestations of infective endocarditis are nonspecific and rare. Endophthalmitis, retinal artery occlusion, Roth spots and vitreal and retinal infiltrations can all be seen with infective endocarditis. Also, infective endocarditis involving the left atrial appendage with no involvement of the mitral valve apparatus is a rarity.

**Case presentation:**

Here we report a case of infective endocarditis of the heart involving the left atrial appendage presenting with features of endogenous endophthalmitis which ultimately progressed to phthisis bulbi with subtle cardiac symptoms in a previous healthy young adult.

**Conclusion:**

Infective endocarditis involving the left chambers of the heart carries an inherent high risk of systemic embolization. Panophthalmitis which is considered to be the most severe form of endogenous endophthalmitis is a rare presenting feature. Although a definitive treatment algorithm is lacking, early surgery and parenteral antibiotics along with local antibiotic injections could help to save the vision.

## Background

Endogenous bacterial endophthalmitis (EBE) is an uncommon intraocular infection caused by hematogenous spread of microorganisms to the eye from extraocular sites, such as the heart, urinary tract or cerebral meninges. Visual disturbance is the most common complaint leading these patients to an ophthalmic evaluation and the diagnosis of ocular involvement. Prompt diagnosis and aggressive treatment are required because EBE is a vision-threatening disease, and its causes are often life-threatening conditions. Infective endocarditis (IE) is a relatively rare disease that is associated with severe complications and a high mortality. The vegetations in IE are typically intra-cardiac and involve the valves. Isolated left atrial mural endocarditis is a very rare condition with only a handful of cases reported in the literature [1]. EBE is a rare presenting complaint of IE. Despite the severity and miserable outcomes of this condition, the best management is still unclear.

## Case presentation

**Fig. 1:**
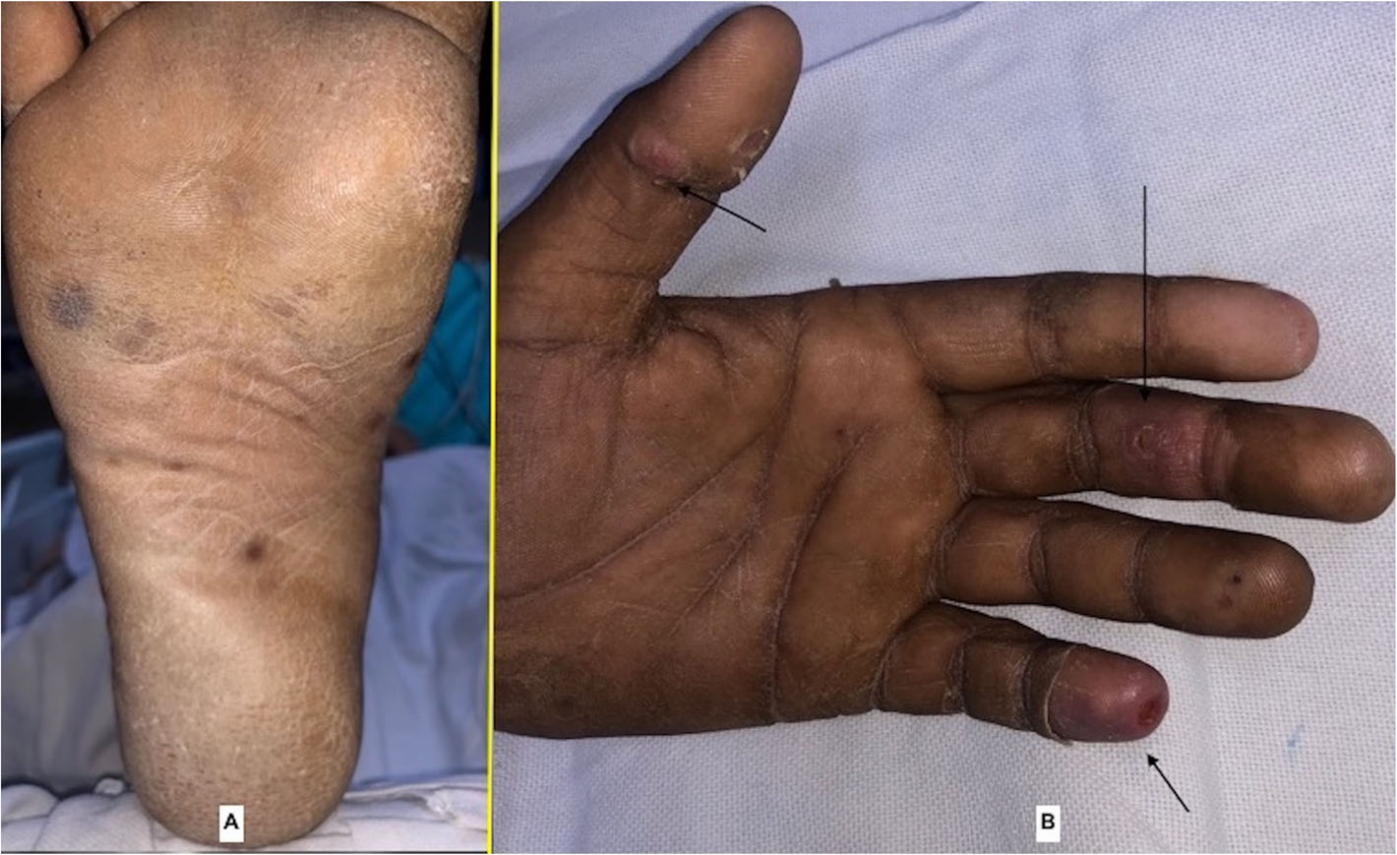
Pre-operative clinical pictures—**A** Janeway lesions on the sole of the foot and **B** Osler nodes on the palm marked by black arrows

**Fig. 2:**
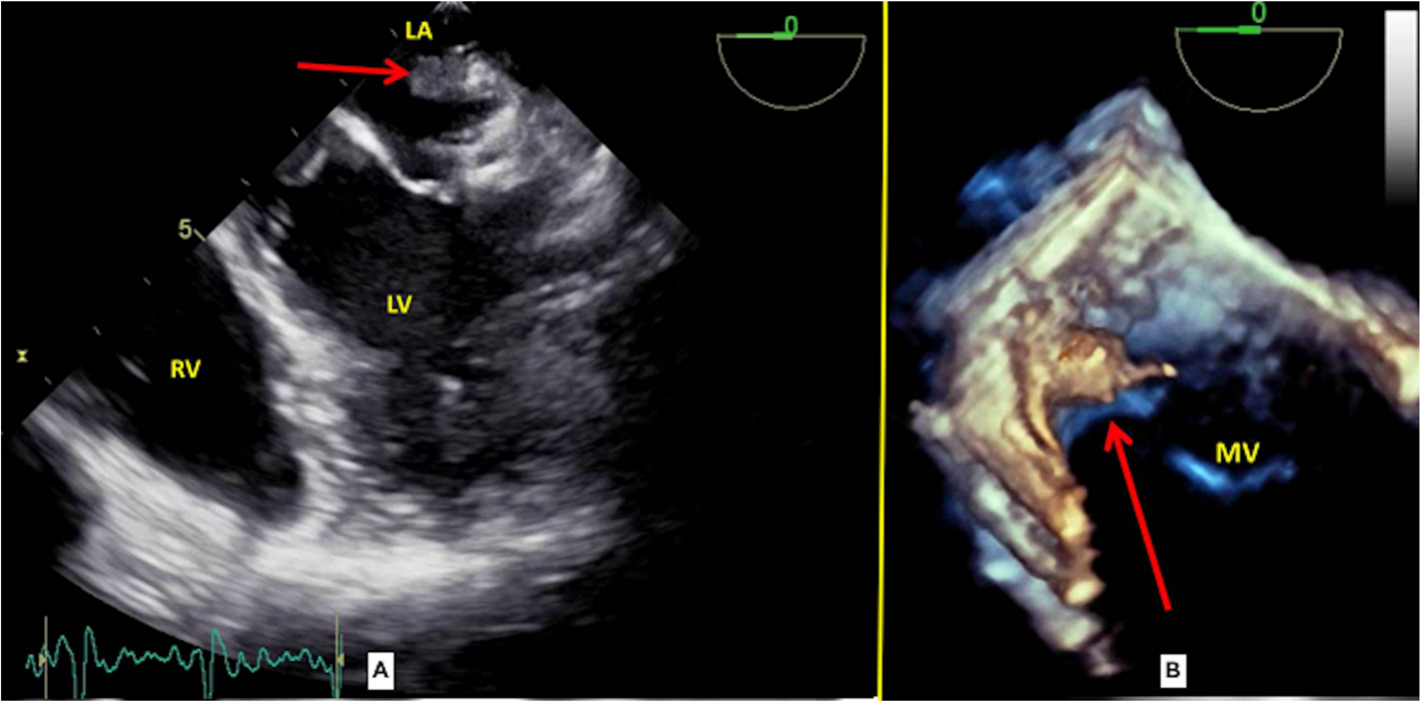
Intra-operative trans-oesophageal echocardiography—**A** vegetation in the left atrium (red arrow) away from the mitral valve and **B** 3D reconstructed image of the same showing the vegetation (red arrow)

**Fig. 3:**
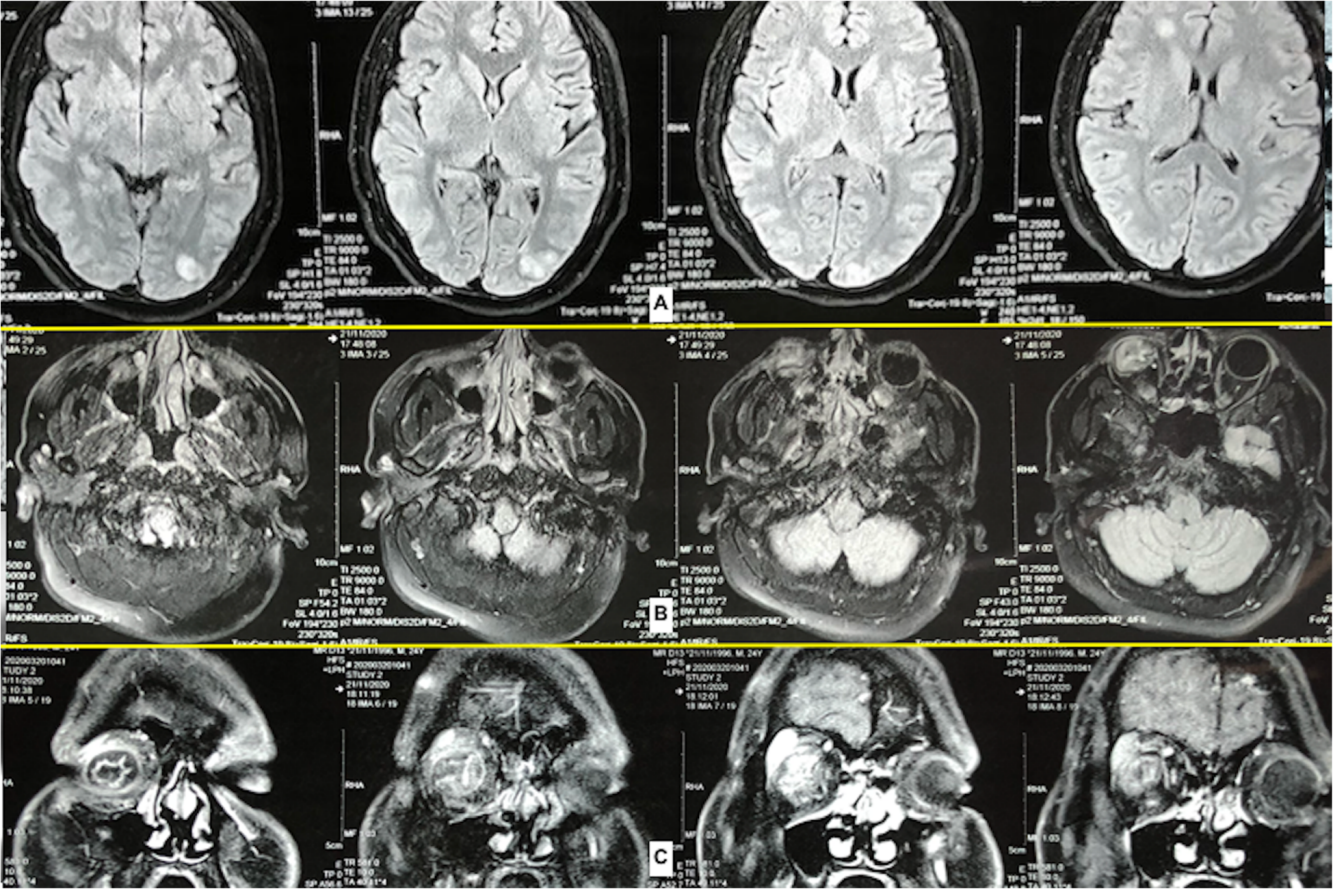
Pre-operative MRI—**A** multiple acute embolic infarcts in the bilateral cerebral hemisphere, **B** sagittal section showing the right globe appears small and distorted with T2 isointense subretinal contents along with thickened retinal and choroidal layers and **C** coronal section showing the same

**Fig. 4:**
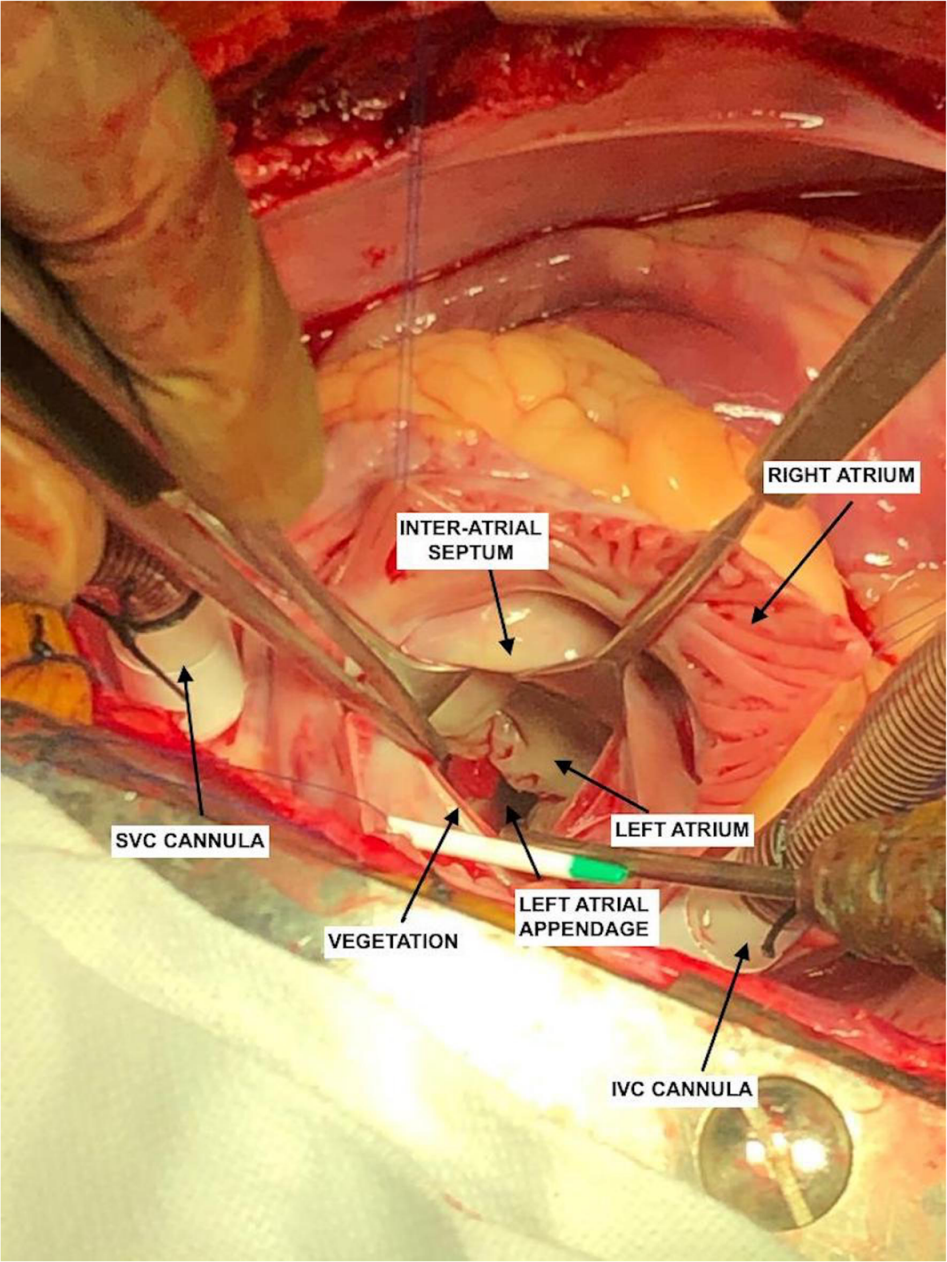
Intra-operative surgical image showing the vegetation

A 24-year-old young adult presented to the emergency with a high-grade fever which was sudden in onset and rapidly progressive, diminished vision and mucopurulent discharge from the right eye for the past 6 days. He had no symptoms of dyspnea, angina or palpitations. He had no history of trauma. On general examination, there were Janeway lesions (Fig. 1A) and Osler nodes (Fig. 1B) present on both hands and feet. There were adhesions in the right eye and he had difficulty in opening his right eye. He was conscious and oriented to time, place and person. Cardiovascular examination was essentially normal with no murmur on auscultation with the exception of tachycardia of 120/min. Routine blood workup and blood cultures were sent as a part of the workup for IE. In view of persistent fever, despite empirical antibiotics over the past 6 days at a local hospital, he was started with intravenous vancomycin (30mg/kg/day) and gentamicin (3mg/kg/day). Blood cultures were positive for *Staphylococcus aureus*, and subsequently, IV antibiotics were changed to vancomycin (30mg/kg/day) and meropenem (3g/day) according to the culture sensitivity. Examination of the right eye revealed conjunctival hyperemia and chemosis and corneal perforation with pus seeping through it. All other systemic examinations were within normal limits. A trans-oesophageal echo was done, which revealed a large mass (6 × 5 mm) attached to a ridge in the left atrial appendage (Fig. 2A, B). Mitral and aortic valves were normal. Ultrasonography of the right eye showed heterogeneous soft tissue density and increased vascularity predominantly in the posterior chamber with subcutaneous oedema of the eyelid muscle. CEMRI of the brain (Fig. 3A) revealed small embolic infarcts in the cerebral hemisphere which were managed conservatively with intravenous antibiotics. CEMRI of the bilateral orbit (Fig. 3B, C) revealed a small and distorted right globe with thickened retinal and choroidal layer and T2 hypointense subretinal contents. The left orbit and its contents were normal. The patient was planned for an urgent surgical excision of the vegetation (Fig. 4) in the left atrium. After median sternotomy and pericardiotomy, the external cardiac anatomy was assessed. RA trans-septal approach was used to assess the vegetation. A large (6 × 5 mm) vegetation was seen at the base of the LA appendage (LAA) which was excised completely. The mitral valve was assessed and was found out to be normal. The LAA was plicated to exclude any further growth of the vegetation and LA was closed following which the patient was weaned off CPB and subsequently reversed with protamine. The patient was shifted to the ICU and extubated after 12 h of mechanical ventilation. The patient was discharged in a clinically stable condition and is now planned for an evisceration of the right eye.

## Discussion

Up to 90% of patients with infective endocarditis present with fever, often associated with systemic symptoms of chills, poor appetite and weight loss. Up to 25% of patients have embolic complications at the time of the diagnosis. Therefore, IE has to be suspected in any patient presenting with fever and embolic phenomena. Imaging, particularly echocardiography, plays a key role in both the diagnosis and management of IE. Echocardiography is also useful for the prognostic assessment of patients with IE, for its follow-up during therapy and after surgery [2]. Three echocardiographic findings are considered as the major criteria in the diagnosis of IE: vegetation, abscess or pseudoaneurysm and new dehiscence of a prosthetic valve [3].

A recent study has shown that conventional trans-oesophageal echocardiography (TOE) underestimates vegetation size and that 3D-TOE (Fig. 2B) is a feasible technique for the analysis of vegetation morphology and size that may overcome the shortcomings of conventional TOE, leading to a better prediction of the embolic risk in IE [4].

Surgical treatment is required in approximately half of the patients with IE because of severe complications. Reasons to consider early surgery in the active phase (i.e. while the patient is still receiving antibiotic treatment) are to avoid progressive heart failure and irreversible structural damage caused by severe infection and to prevent systemic embolism [5]. Embolic events are a frequent and life-threatening complication of IE related to the migration of cardiac vegetations. The brain and spleen are the most frequent sites of embolism in left-sided IE, while pulmonary embolism is frequent in native right-sided and pacemaker lead IE. The eye is a rare site for embolism in IE. Also, common risk factors for the development of endocarditis such as valvular heart disease, intravenous drug use or prior endocarditis were not present in our patient.

The period from systemic to ocular signs in EBE is short. The rapid manifestation of ophthalmic symptoms from the onset of sepsis is associated with a poorer prognosis [6] and may serve as a marker of a high virulence of the bacteria. An individual with persistent high-grade fever not responding to antibiotics should have a high suspicion of IE mandating cardiovascular examination and echocardiography to exclude any vegetation.

## Conclusion

Infective endocarditis involving the left chambers of the heart carries an inherent high risk of systemic embolization. Panophthalmitis which is considered to be the most severe form of endogenous endophthalmitis is a rare presenting feature. Although a definitive treatment algorithm is lacking, early surgery and parenteral antibiotics along with local antibiotic injections could help to save the vision.

## Data Availability

Not applicable

## Abbreviations

CEMRI: Contrast-enhanced magnetic resonance imaging
CPB: Cardiopulmonary bypass
EBE: Endogenous bacterial endophthalmitis
ICU: Intensive care unit
IE: Infective endocarditis
LAA: Left atrial appendage
LA: Left atrium
RA: Right atrium
TOE: Trans-oesophageal echocardiography
